# A Letter from Paris

**Published:** 1875-06-15

**Authors:** 


### A LETTER FROM PARIS.

EDITORS MEDICAL EXAMINER: As the result of the last concours of agregation in medicine, MM. Dieulafoy, Grancher, Lionville, Lepine, and Legroux have become agreges of the Faculty of Paris, and MM. Grasset and Balestre of the Faculty of Montpellier. The term agrege conveys the meaning of "fellow," or, as used with us, might, perhaps, be better translated as assistant professor. From the agreges are elected the professors, as vacancies occur in the Faculty.

The road to this position is by no means an easy one to travel, and this system of competitive examinations is one that the profession in France is justly proud of. The concours is open to any graduate of the French school who has reached the age of twenty-five years. For the nine vacancies of this last concours there were twenty candidates.

The first trial was an essay upon Anatomy and General Physiology, and the subject given was "On the Lymphatic System." This had to be treated without notes or aid of books for reference, and each candidate read his essay before the committee of the Faculty, in the large amphitheatre of the school, which of course was open to the public.

The second examination consisted in a lecture of three-quarters of an hour on some given medical question, three hours being granted for preparation, but without aid of books or notes. The jury then having examined the printed works of each candidate, the opening trial was finished and two of the competitors were eliminated.

Then commenced the final trials (*epreuves definatives*). The first was a public lecture of an hour, on a subject of general pathology given by the jury twenty-four hours previously. The second consisted in a public clinical lecture on some case, designated by the jury, in the hospital wards (Hotel Dieu or Pitie), after fifteen minutes' examination by the candidate. The third and last trial was the writing of a thesis (subject given by the jury), which had to be *printed* in regulation form seventeen days after the subject was announced.

From this summary of the requirements of the concours of agregation, it will be seen that it is no easy matter to acquire the title of agrege, and that there is no "royal" road to that position. Although the concours was opened in December, the theses were not finished until April. As all the examinations were public, they were followed very closely, not only by the students but by the graduates of the school, for there is always an interest felt in these contests by the profession at large, and there are few things connected with the school in which the French take more pride than in these struggles for position. There is no chance for favoritism in the examinations, as their publicity forbids any underhand work. One curious part of the concours is the "quizzing" of one candidate by the others. It is upon the thesis. The candidate seats himself in the "pulpit" of the large amphitheatre, thesis in hand; while, seated at a table in front of him, are the members of the jury, attired in their red silk gowns and hats, and two of the competitors, likewise supplied with copies of the thesis. The latter successively ply the "man in the pulpit" with questions on the subject of his thesis, trying to break down any theory that he may have advanced, or to catch him upon some point that he may not have considered. The one who questions is incited, perhaps, by the thought that he soon will undergo the same process, or, indeed, he may be smarting under the remembrance of questions that were given to embarrass him in the presence of eight or nine hundred auditors. With that characteristic French style, the questioner generally commences with a few remarks upon the pleasure he has had in reading the essay before him, and congratulates the author upon its merit, and then he suddenly launches out upon some hidden question that does not always betray the amount of good feeling one would expect after his prorogation. The author of the thesis commences his reply by thanking the other for his congratulations, perhaps expressing himself as highly honored by such a compliment from so distinguished a confrere, and then replies to the questions asked, referring, it may be, to another page of his thesis where the answer may be found, or, perhaps, giving some new references to prove his position. Thus it goes on, and the jury and public sit and listen, generally amused at the contest that is going on before them. One can judge that under these circumstances a man's ability has a clear chance of making itself evident, although there is in such trials, perhaps, a tendency to the cultivation of egotism, which may in some measure account for peculiarities of to-day.

The report of M. Chauffard, president of the jury, to the Minister of Public Instruction, mentions this concours as the most satisfactory that has been held for many years, and pays high compliments to those who were not elected as well as to the successful ones. The subjects for the theses are chosen by the Faculty with reference to those questions that are considered as needing more research, and we find the pathology of the nervous system occupying a prominent position among the subjects given. Five subjects were on questions of special and general therapeutics. This last choice was not amiss, for therapeutics are not too closely investigated here. Even the writer in the half-official *Gazette des Hopitaux* admits this, for in this connection we find the following rather pointed inquiry: "Does the Faculty commence to understand that therapeutics is the feeble side of its teaching, and that, without losing anything for the future in its useful instruction on pathological anatomy and pathogeny, it is time to direct its studies a little toward that which is the object and goal of all medical researches?" This is rather a broad hint, but unfortunately embodies too much truth in indicating a very weak point in French medical training. To forget the patient in studying his disease is, to speak mildly, rather an awkward thing for the patient.

Dr. Siredey has published an article in the March and April numbers of the *Annales de Gynecologie*, which is entitled: "Puerperal Fever does not Exist." He holds that under the name of puerperal fever are confounded many different diseases, each having a proper train of symptoms, a special course, and a variable prognosis, and that among them it is possible to make a differential diagnosis. The numerous and varied opinions that are held upon this question he ascribes to the variety of lesions that have been found upon *post-mortem* examination. For some persons the metritis embodies the principal lesion, while for others a peritonitis, phlebitis, or lymphangitis has the preference. The essentialists object that the disease cannot be named from any of the lesions, as these vary with the case, with the epidemic, or indeed may be altogether wanting. Dr. Siredey contests this last view that there may be no lesion, and holds that the autopsies were not made with sufficient care, and are too few to be of value. In support of this opinion he mentions several cases in which, though at first nothing abnormal was found, a more careful examination demonstrated the presence of pus. From the vast number of cases examined where lesions were found, and from the few and doubtful records to the contrary, he deems it just to admit that the fever is not essential. Nevertheless, the name "puerperal fever" is preserved, and the various lesions in different parts of the body are considered as only secondary manifestations of this one disease. It matters little whether the action turns itself upon the uterus, the peritoneum, the veins, or the lymphatics; for the upholders of this idea, it is sufficient to know the ætiological conditions of the disease to be sure of its nature. So constituted, puerperal fever has been accepted by the most eminent. Depaul, P. Dubois, and Danyau, in the discussion of 1858, defended it. Trousseau, at first doubting, finally admitted it, not only in women, but in infants, and, indeed, in wounded men, provided they were placed near an obstetrical service! In spite of his respect for such eminent authorities, Dr. Siredey believes that in retaining the name "puerperal fever" we only perpetuate an error, and obscure a question that it is of the highest importance to clear up. In fevers we find certain lesions that are constant. For example, in typhoid fever there is the ulceration of Peyer's patches, in scarlet fever the serous membranes are attacked, and the mucous in measles. For the different lesions that are found after death from the disease called puerperal fever, Dr. Siredey thinks he is able to point out a series of symptoms that will show that several distinct diseases have been confounded heretofore, and that a differential diagnosis can be made among them. After mentioning the peritonitis, with its typhoid state and fetid diarrhœa and lochia, he treats of the really difficult, and for many impossible, diagnosis between phlebitis and lymphangitis. He commences by endeavoring to show that many times the lesions considered to be in the veins were in the lymphatics of the uterus. The principal trunks of the lymphatics occupy the angles and sides of the uterus, and sometimes they acquire a volume of 5, 6, or 8 millimetres, equaling that of the veins, and hence liable to be confounded with them. The large circular vessel, situated at the juncture of the neck and body of the uterus, which is so often found filled with pus, is not a vein, but a lymphatic. Dr. Siredey thinks that many mistakes have been made from these anatomical peculiarities, and considers that inflammation of the lymphatics is the more common. Noting the very intimate connection of the lymphatics with the peritoneum, and the frequent occurrence of peritonitis in the fever of child-bed, he argues that the so-called puerperal fever is generally but a lymphangitis. The differential diagnosis between this disease and phlebitis he places as follows: Chill—In the lymphangitis it is unique, and only appears at the commencement of the disease, while in phlebitis it is multiple, and not only is found at the beginning but at all periods of the disease. The chill of the lymphangitis appears in general from the first to the fifth day after the labor, while in an inflammation of the veins it occurs but rarely before the sixth day, and frequently much later. The temperature reaches a high figure (40° to 41° C.) in the lymphangitis, but remains at this level, varying only by some tenths of a degree. In the phlebitis the temperature rises progressively, reaching its maximum during the febrile paroxysm; then falls to rise again, presenting between the remissions and exacerbations a difference of several degrees. The pain is more intense in the abdomen for the lymphangitis, which may be accounted for by the peritonitis. The phlebitis does not give rise to intense abdominal symptoms, but reveals its existence by varied lesions that testify to the invasion of the economy by pyæmia. The course of disease is much more rapid in the lymphangitis. Death occurs generally eight or ten days after the labor, and in rare cases, in two or three days, while the phlebitis may extend from two to three weeks. Dr. Siredey admits that lesions of both diseases are found in some cases, but states that the symptoms show the first period belongs to the lymphangitis, while the second period is that of phlebitis. He illustrates his argument by giving in detail the history of four cases.

During the past two weeks the Paris journals, medical and secular, have been very much occupied with the voyage of the balloon Zenith. Messrs. Croce-Spinelli, Sivel and Tissandier made the ascent on the 15th of April, and gained the height of 8,600 metres, but unfortunately the two former died during their exposure in this rarefied atmosphere, and Tissandier alone remained to tell of their experience. The ballon was equipped with all the inventions that science has shown to be of use in such voyages, and each person was provided with a bag of oxygen by means of which he could supply himself with that gas when the quantity in the air should become too limited. However, these precautions seem to have been of but little service. Tissandier states that at twenty minutes after one o'clock (two hours after they left the earth) he saw the needle of the barometer pass 280 millimetres, and he tried to say "We are at 8,000 metres," but was unable to speak. The balloon then descended a short distance, but soon mounted again, and all three lost consciousness. When Tissandier came to himself, he found the balloon falling with frightful velocity and his two companions dead. Self-registering tubes had been placed in the balloon to note the atmospheric pressure, and, though the majority of them were broken, two indicated that the maximum height reached must have been 8,600 metres. The question that greatly interests the scientific world is concerning the cause of the death of Croce-Spinelli and Sivel. Experiments have frequently been made in Paris by means of an air-tight chamber, in which the pressure of the atmosphere can be reduced as desired. Under these couditions, M. Bert has been able, by inhaling oxygen, to support a diminution corresponding to an ascent of 8,900 metres. But unfortunately the conditions of the experiment and of an ascent have not been placed on exactly the same level. The subject is at present under consideration at the Academy of Medicine, but the opinion, as found in the medical journals, seems to be that the death was the result of an asphyxia from privation of oxygen, with the added influence of excessive cold; and, secondary to this, to pulmonary lesions such as accompany the asphyxia from carbonic acid. It is thought that the voyagers were not sufficiently careful in supplying themselves with the oxygen, or that the rapidity of the ascent prevented them from using the gas. One result of this balloon trip is an outcry against researches in such high regions, and the Academy finds itself called upon to discourage any future attempts by not offering inducements for the venture. Of course the wide-spread interest in this fatal attempt has incited all those with a "ballooning" turn of mind, and already we hear of new ascents that are to take place.

It is generally admitted that Paris leads the world for fashion, and I doubt not there is a great deal of truth in such an assertion. More particularly was I convinced of this when I heard the following *fashionable* indication for the early use of the forceps: When a public singer is brought to the pangs of child-bed, one must use the forceps as soon as possible, lest the continued screaming of the woman should injure her voice. C. C. M.

PARIS, May 3d, 1875.

---

CHLORAL IN TETANUS. — In the treatment of traumatic tetanus by chloral, Dr. Gontier reaches the following conclusions: Chloral may render great service in the treatment of chronic or subacute tetanus, and is especially preferable to other drugs. It is completely inefficacious in acute tetanus, and only has a slight palliative action. It may be advantageously associated with tonics, diffusible stimulants, and diaphoretics. Intravenous injections of chloral are extremely dangerous, and should, in the present state of science, be reserved for exceptional cases only. — *Phil. Med. Reporter*.

THE DURATION OF LIFE.—The following facts on the duration of life appear in the *Deutsche Versicherungs Zeitung*:

"In ancient Rome, during the period between the years 200 and 300 A. D., the average duration of life among the upper classes was 30 years. In the present century, among the same classes of people, it amounts to 50 years. In the sixteenth century the mean duration of life in Geneva was 21.21 years; between 1814 and 1833 it was 40.68 years; and at the present time as many people live to 70 years of age as 300 years ago lived to the age of 43."—*Ibid.*

